# *Tm7sf2* Disruption Alters Radial Gene Positioning in Mouse Liver Leading to Metabolic Defects and Diabetes Characteristics

**DOI:** 10.3389/fcell.2020.592573

**Published:** 2020-11-23

**Authors:** Leonardo Gatticchi, Jose I. de las Heras, Aishwarya Sivakumar, Nikolaj Zuleger, Rita Roberti, Eric C. Schirmer

**Affiliations:** ^1^Department of Experimental Medicine, University of Perugia, Perugia, Italy; ^2^Institute of Cell Biology, University of Edinburgh, Edinburgh, United Kingdom

**Keywords:** nuclear envelope, Tm7sf2, NET47, genome organization, tissue specificity

## Abstract

Tissue-specific patterns of radial genome organization contribute to genome regulation and can be established by nuclear envelope proteins. Studies in this area often use cancer cell lines, and it is unclear how well such systems recapitulate genome organization of primary cells or animal tissues; so, we sought to investigate radial genome organization in primary liver tissue hepatocytes. Here, we have used a *NET47/Tm7sf2^–/–^* liver model to show that manipulating one of these nuclear membrane proteins is sufficient to alter tissue-specific gene positioning and expression. Dam-LaminB1 global profiling in primary liver cells shows that nearly all the genes under such positional regulation are related to/important for liver function. Interestingly, Tm7sf2 is a paralog of the HP1-binding nuclear membrane protein LBR that, like Tm7sf2, also has an enzymatic function in sterol reduction. *Fmo3* gene/locus radial mislocalization could be rescued with human wild-type, but not *TM7SF2* mutants lacking the sterol reductase function. One central pathway affected is the cholesterol synthesis pathway. Within this pathway, both *Cyp51* and *Msmo1* are under Tm7sf2 positional and expression regulation. Other consequences of the loss of Tm7sf2 included weight gain, insulin sensitivity, and reduced levels of active Akt kinase indicating additional pathways under its regulation, several of which are highlighted by mispositioning genes. This study emphasizes the importance for tissue-specific radial genome organization in tissue function and the value of studying genome organization in animal tissues and primary cells over cell lines.

## Introduction

Many gene loci important for tissue functions move away from the nuclear envelope (NE) as they become activated during differentiation (Kosak et al., 2002; Williams et al., 2006; Morey et al., 2008; Szczerbal et al., 2009), whereas other genes inhibitory to differentiation move from the interior to the NE as they are shut down (Peric-Hupkes et al., 2010; Robson et al., 2016). For example, the *Mash1* locus moves from the NE in neural progenitor cells to the nuclear interior during neuronal differentiation concomitant with its activation and loses silencing epigenetic marks in the process (Williams et al., 2006). In the opposite direction, *Nid1*, a gene needed for early stages of myotube fusion that later becomes inhibitory, moves to the NE during myogenesis and is shut down (Robson et al., 2016). Understanding the full impact of this additional level of regulation from radial repositioning has been impeded by the inability to specifically disrupt the positioning of endogenous genes and the virtual absence of animal models in this area of research. Nonetheless, its importance is underscored by links to human disease. For example, one study recapitulated a human developmental disorder in mice by genome deletions that disrupted the formation of local topological domains and the ability to establish long-scale enhancer–promoter interactions (Lupianez et al., 2015), whereas another found point mutations in previously unlinked Emery-Dreifuss muscular dystrophy patients occurring in muscle-specific NE transmembrane proteins (NETs) that block their function in establishing tissue-specific patterns of radial genome organization (Meinke et al., 2019).

We previously identified several tissue-specific NETs that direct tissue-specific patterns of radial genome organization (Korfali et al., 2010; Zuleger et al., 2013; Robson et al., 2016; de las Heras et al., 2017). This, for the first time, allowed knockdown of a single protein to disrupt just the subset of tissue-specific NE–genome interactions. For example, knockdown of muscle-specific NET39 prevented the normal repositioning to the NE during myogenesis of the *Nid1*, *Ptn*, *Msc*, *Vcam1*, and *Bdnf* genes as confirmed by fluorescence *in situ* hybridization (FISH) and dozens more as indicated by DamID, a technique used to identify chromatin positioned at the nuclear periphery (Vogel et al., 2007); moreover, repositioning was associated with partial de-repression compared with wild-type (WT) (Robson et al., 2016). At the same time, overexpression of this NET in myoblasts, where these genes are normally expressed and in the nuclear interior, resulted in their recruitment to the NE in the absence of myogenesis; however, unlike their disrupted repositioning to the periphery in myotubes, this altered positioning did not affect *Nid1* expression. This suggests that the full effects of changes in spatial genome organization require additional factors only present in the tissues where a particular pattern of genome organization is manifested. Accordingly, we were interested to test if genome organization changes and their functional consequences differed in actual animal tissues.

To do so, we turned to a mouse knockout (KO) of NET47/Tm7sf2 (Bennati et al., 2008). TM7SF2 is a NET that we previously demonstrated contributes to normal peripheral positioning of some chromosomes in human cells (Zuleger et al., 2013). TM7SF2’s ability to influence genome organization is consistent with its having a subcellular pool in the NE in several cell types, though it has a predominant pool in the endoplasmic reticulum (ER) in most cell types tested (Roberti et al., 2002; Schirmer et al., 2003; Malik et al., 2010; Korfali et al., 2012; Capell-Hattam et al., 2020). TM7SF2 is highly preferentially expressed in the liver among human tissues (Zuleger et al., 2013), and the mouse Tm7sf2 is > 40-fold higher than the median value for its expression in 96 mouse tissues with the next highest tissue, dorsal root ganglia, at just 13-fold^1^ (Wu et al., 2009). Accordingly, its function in genome organization is relatively specific to the liver. For example, human chromosome 5 is normally peripheral in liver cells; however, it is located in the nuclear interior of kidney cells where the expression of TM7SF2 is negligible (Zuleger et al., 2013). Similarly, knocking down TM7SF2 in a human liver cell line relocated the chromosome to the nuclear interior. Compellingly, TM7SF2 can also affect genome organization when exogenously expressed in the heterologous HT1080 cell line, and it preferentially affects the radial positioning and *de novo* expression of liver genes even though the cell line is derived from a fibroblast tumor (de las Heras et al., 2017). Interestingly, the liver-specific TM7SF2 yielded gene changes in the HT1080 fibroblasts mimicking some of the changes in gene expression that occur in hepatic differentiation, whereas muscle-specific NET39-induced HT1080 gene changes favored inhibition over activation of hepatic genes. These results together indicated that while the transcriptional and chromatin-repositioning changes induced by NETs require other factors for successful differentiation, NETs direct some of the differentiation-associated changes in the absence of differentiation, and even in completely unrelated cell types.

TM7SF2 is a paralog of the well-characterized NET lamin B-receptor (LBR) that has been linked to the human disorders Pelger-Huet anomaly (Hoffmann et al., 2002) and Greenberg skeletal dysplasia (Waterham et al., 2003). LBR has an important genome-organizing function from its ability to directly bind heterochromatin protein 1 (HP1) (Ye and Worman, 1996) and its interaction with generally repressive chromatin at the NE (Makatsori et al., 2004). The relevance of this function has been shown by combined knockdown of LBR and lamin A altering heterochromatin distribution in certain cell types (Solovei et al., 2013), potentially relevant in the context of the tendency for silenced chromatin to be at the NE (Pickersgill et al., 2006; Pindyurin et al., 2007). Although the mapped HP1-binding sites for LBR are in the N-terminal region that differs from that of Tm7sf2 (Ye et al., 1997), this does not discount the possibility of Tm7sf2 having different chromatin-binding partners. At the same time, both LBR and Tm7sf2 have 3beta-hydroxysterol Delta(14)-reductase activity (Silve et al., 1998; Bennati et al., 2006) that could potentially contribute indirectly to their genome organization effects and the LBR disease pathomechanism. Livers from the *Tm7sf2^–/–^* mice are morphologically indistinguishable from those of WT mice and do not show reduced cholesterol presumably due to the ability of LBR to suffice for this function (Bennati et al., 2008). However, the mice exhibit metabolic deficiencies resulting in the accumulation and delayed catabolism of hepatic triglycerides, and this with delayed cell cycle progression after hepatectomy contributes a persistent steatosis (Bartoli et al., 2016). Thus, there were many potential mechanisms by which Tm7sf2 might exert its effects, ranging from very specific gene interactions to affecting more generally the distribution of heterochromatin in these cells, or indirect effects from its sterol reductase functions. Interestingly, many genes in cell cycle pathways were altered in the KO animals, suggesting that a primary role in genome organization might underlie the cell proliferation defect (Bennati et al., 2008).

Here, we applied DamID in hepatocytes obtained from livers of WT and *Tm7sf2^–/–^* mice to globally identify genes at the NE. Though only a subset of repositioning genes exhibited corresponding changes in expression, nearly all those under this type of positional regulation were important genes for liver function. Such confirmed regulated genes included *Fmo3* and several others involved in xenobiotic metabolic processes, *Cyp51* and *Msmo1* and several other genes involved in cholesterol production and regulation, and the *Irs1/2* and other genes involved in insulin regulation. As Tm7sf2 also has an enzymatic function in sterol reduction, we tested for rescue with WT and enzymatically dead mutants, finding that the repositioning activity depends on its sterol reductase function. While this is a very functionally interesting result, it also has the negative effect of making more difficult to determine if a phenotype in the mouse is due to its sterol reductase function or its genome-organizing function. However, several defects in the KO mice including weight gain, insulin sensitivity, and a big drop in levels of active Akt kinase correlate with genes changing in position and so are likely due to genome organization defects.

## Materials and Methods

### Animal Phenotyping

*Tm7sf2^+/+^* (WT) and *Tm7sf2^–/–^* mice (Bennati et al., 2008) on a C57BL/6 background were maintained in a temperature-controlled room, under a 12 h light/12 h dark cycle, and given *ad libitum* access to food and water. All experimental procedures were approved by the Animal Care and Use Committee of Perugia University and the Italian Ministry of Health and carried out in accordance with the European Directive (2010/63/EU). Body weight and food intake assessments were performed in 1-year old WT and *Tm7sf2^–/–^* mice fed with a standard chow diet (Mucedola, 4RF21, 3.8 kcal/g, 18.5% calories from protein, 3% from fat, and 46% from carbohydrate).

### Insulin and Glucose Tolerance Tests

For insulin and glucose tolerance tests, mice were allowed to acclimatize to the procedural room for 1 h between 9 and 10 am, and tests were performed at 10 am. Animals were injected intraperitoneally with insulin (1 mU/g) or glucose (1 g/kg). A fixed dose of insulin and glucose was given to all mice in the study group based on the average weight of the group. Blood samples were collected by tail nick at 0, 15, 30, 60, and 120 min, and glucose excursion was measured using the Accu-Chek guide (Roche).

### Immunoblotting of Liver Homogenates and Membrane Fractions

Liver extracts were prepared by homogenization of liver tissues in 19 volumes of phosphate buffered saline (PBS) (w/v) containing phenylmethylsulfonyl fluoride (PMSF, 1 mM) and phosphatase inhibitors sodium fluoride (10 mM) and sodium orthovanadate (1 mM) by sonicating for 10 s 5 × on ice. Liver lysates were centrifuged for 1 min at 13,000 × *g*, and the supernatant was recovered for protein content assay and then denatured by boiling in the presence of Laemmli buffer. For total membrane fraction isolation, liver lysates were subjected to ultracentrifugation at 100,000 × *g* for 60 min. Total membrane pellets were resuspended in 0.25 M sucrose, 2 mM 2-mercaptoethanol, and 0.03 mM PMSF. Protein concentrations were measured by Bradford assay (Bio-Rad) using bovine serum albumin (BSA) as standard. Total liver extracts (60 μg) or membrane proteins (30 μg) were separated on sodium dodecyl sulfate (SDS)-polyacrylamide gels and transferred to nitrocellulose or polyvinylidene fluoride (PVDF) membranes, respectively, at 100 V for 90 min. Filters were probed with the following primary antibodies: p-Akt Ser^473^ (1:1,000, 9,271) and total Akt (1:1,000, 9,272) by Cell Signaling Technology; Gapdh (1:500, sc-32233) by Santa Cruz Biotechnology; Srebp-2 (1:1,000, ATCC^®^ CRL2545) was a kind gift from Prof. T. Osborne to Rita Roberti; Tm7sf2 (1:1,000) (Roberti et al., 2002), Hmgcr (1:500, 3,952), and Ldlr (1:1,000, 3,839) by BioVision; and horseradish peroxidase (HRP)-conjugated secondary antibodies (1:5,000, Bio-Rad). Chemiluminescence was visualized using the ECL reaction, and images were acquired by the ChemiDoc XRS+ imaging system. Densitometric analysis was performed using the Quantity One software (Bio-Rad).

### Lentivirus Construction and Generation

DamID lentiviral constructs were a gift from Bas van Steensel and are previously described (Vogel et al., 2007). For rescue experiments, Tm7sf2 with a C-terminal EGFP tag was cloned by moving the gene from pEGFP-N2 (Malik et al., 2010) into pRRLSIN.cPPT.PGK-GFP.WPRE lentiviral vector (Addgene #12252). *TM7SF2* point mutations were obtained from studies on C14SR homologs (Prakash and Kasbekar, 2002; Li et al., 2015) and generated in the lentiviral vector by site-directed mutagenesis. All lentiviruses were then generated from these constructs using standard techniques as previously described (Gatticchi et al., 2019).

### Isolation, Culture, and Lentiviral Transduction of Primary Mouse Hepatocytes

Primary hepatocytes were isolated from 12- to 16-week old WT and *Tm7sf2^–/–^* mice by a two-step collagenase perfusion method (Gatticchi et al., 2019). For transduction experiments, primary hepatocytes were cultured in collagen-coated 6-well plates with 0.8 ml serum-free William’s E medium supplemented with 2 mM L-glutamine, 100 U/ml penicillin, 0.1 mg/ml streptomycin, 100 nM dexamethasone, 100 nM insulin, and 40 ng/ml epidermal growth factor (EGF) and then after 4 h incubated for 24 h with 100 μl of lentiviral vectors encoding for Dam alone, Dam-LaminB1, or empty vector, all in the presence of a non-toxic dose of the transduction enhancing agent polybrene (6 μg/ml) in a 5% CO_2_/water-saturated incubator at 37°C. On the next day, the medium was discarded and renewed with fresh medium daily. After 60 h following transduction, cells were harvested for genomic DNA isolation. Cells were checked for mycoplasma contamination before use (Robson and Schirmer, 2016). In rescue experiments, *Tm7sf2*-KO primary hepatocytes were transduced for 24 h with lentiviral vectors encoding for WT TM7SF2 or mutants and either fixed with paraformaldehyde (PFA) (4% in PBS) 60 h after transduction for FISH analysis or taken at 48 or 72 h after transduction for RNA isolation.

### Microarray Analysis

Transcription data for livers from WT and *Tm7sf2^–/–^* mice were previously obtained using the Affymetrix MOE430 v2.0 platform (Bennati et al., 2008). We reanalyzed the data using the BioConductor package Limma (Ritchie et al., 2015) after applying quantile normalization. Differentially expressed transcripts were selected adjusting for a false discovery rate (FDR) of 5% and a fold-change cutoff of 1.4 (abs[log_2_(KO/WT)] = 0.5). For transcription factor (TFs) analysis only, we relaxed the parameters by omitting the fold-change cutoff. Raw and processed microarray data from the current analysis were deposited at NCBI GEO with ID GSE155762.

### DamID

DamID is a technique that can be used to identify chromatin at the nuclear periphery (Vogel et al., 2007). It uses a vertebrate LaminB1 protein, an abundant protein of the nuclear lamina, fused to a bacterial Dam methylase, which methylates adenosines found in a GATC tetranucleotide sequence context, a type of methylation not found in mammalian genomes. Dam-methylated sequences can then be selectively amplified by PCR and identified by DNA sequencing. For full details, see Gatticchi et al. (2019) and Robson and Schirmer (2016). Briefly, genomic DNA from hepatocytes transduced with Dam-LaminB1 or dam control was purified using a Qiagen DNeasy tissue lysis kit (Qiagen, 69504) as per the manufacturer’s instructions. DNA methylated by the dam methylase was then digested with *DpnI*, which cuts into the GATC sequence when methylated. Next, specific adaptors were ligated, and the DNA was subjected to digestion with *DpnII* that recognizes unmethylated GATC sequences. This digestion ensures that the only fragments that can be amplified using the specific adaptors are those flanked by methylated GATC motifs and lacking any internal unmethylated GATC. The DNA was then sequenced (Illumina HiSeq 2000, Beijing Genomics). Sequence reads were mapped to the mouse mm9 genome and the log_2_(Dam-LaminB1 / dam control) determined for all genomic *DpnI* fragments. Lamina-associated domains (LADs) were determined using the peak finder function in the BioConductor package DNAcopy (Seshan and Olshen, 2016) using the default parameters as described by Robson et al. (2017). Regions displaying differential nuclear lamina association between the WT and the KO hepatocytes were identified by subtraction of LADs. We indicate the direction of repositioning of these differential regions (DRs) naming them PI (indicating peripheral association in the WT that is lost, becoming more internal, in the KO) or IP (indicating regions that are recruited to the nuclear periphery in the KO). We then classified every gene according to how their radial position changes in the KO mouse liver. When a gene overlapped with an IP or a PI region, the genes were also referred to as IP or PI. Some genes overlap both PI and IP regions, and those were flagged with the name AMB for ambiguous. Genes that stayed at the NE were named PP, and genes that stayed internal were named II.

DamID data are available at NCBI GEO with ID GSE155762.

### Functional Analysis of Gene Sets

Functional analysis of gene sets was performed with the BioConductor package topGO (Alexa and Rahnenfuhrer, 2019) using Gene Ontology Biological Process categories. TFs and their targets were obtained from the TRRUST database version 2^2^. The list of genes differentially expressed at a FDR of 5%, without fold-change cutoff, was checked for TFs and their targets extracted. This information was used to identify what proportion of differentially expressed genes were likely a result of their upregulated TFs, and not the result of repositioning. Further search for genes with transcriptional regulation activity was performed using Gene Ontology (GO) data^3^, namely, GO categories: GO:0008134 (TF binding), GO:0005667 (TF complex), GO:0006355 (regulation of transcription, DNA-templated), GO:0003700 (DNA-binding TF activity), and GO:0003713 (transcription coactivator activity).

### RNA Isolation, PCR, and Quantitative RT-PCR

Total RNA isolation, cDNA synthesis, and quantitative real time-PCR (qRT-PCR) were performed as previously described (Gatticchi et al., 2017). The mRNA expression levels of target genes were normalized to *Hprt* expression. To test the expression of WT *TM7SF2* and mutants in rescue experiments, PCR was performed in a final volume of 20 μl with 25–50 ng of cDNA template and using JumpStart^TM^ REDTaq^®^ ReadyMix^TM^ (Sigma-Aldrich) according to the manufacturer’s instructions. The cycle program consisted of an initial denaturation step of 3 min at 95°C, then 26 cycles of 30 s at 95°C, 1 min at 60°C, and 30 s at 72°C, with a final extension step of 1 min at 72°C. PCR products were fractionated through 1.5% agarose gel stained with 1 μg/ml ethidium bromide, and images were acquired by a ChemiDoc XRS+ imaging system. Primers used for qRT-PCR are listed in Table 1.

**TABLE 1:** List of primers for qRT-PCR.

Gene	Forward primer	Reverse primer
*Mouse*		
*Abca1*	CCAGACGGAGCCGGAAGGGT	GTGCCCATGTCCTCGGGAGC
*Cyp51*	ACGCTGCCTGGCTATTGC	TTGATCTCTCGATGGGCTCTATC
*Fmo3*	GGAACTTGCACTTTGCCTTC	TAGGAGATTGGGCTTTGCAC
*Hmgcr*	TGCCTGGATGGGAAGGAGTA	GCCTCGAGTCATCCCATCTG
*Hprt*	GAGAGCGTTGGGCTTACCTC	ATCGCTAATCACGACGCTGG
*Irs1*	TCCAGAAGCAGCCAGAGGA	AGGATTTGCTGAGGTCATTTAGGT
*Irs2*	GCCTACACGCCTATCGCTAGA	CTCTTGGGCTCTGTGGGTAGA
*Lbr*	GTGCTCCTGAGTGCTTAC	GCCAATGAAGAAGTCGTAG
*Ldlr*	GGGAACATTTCGGGGTCTGT	AGTCTTCTGCTGCAACTCCG
*Msmo1*	ACCATACGTTTGCTGGAAACCATC	AGCGCCCGTATAAAAAGGAACCAA
*Nsdhl*	GACACATCTTAGCCGCTGAGCAC	CAGAAAGGGATTGGTTCATCGTT
*Srebp2*	CAAGCACACTGATTGAGAT	TGGGCACGATTTAAGAAGTA
*Vldlr*	GAGCCCCTGAAGGAATGCC	CCTATAACTAGGTCTTTGCAGATATGG
*Human*		
*TM7SF2*	TGGCTTCCAGTTGCTCTACG	TGAAGCCAAACCCGTCATGT

### Fluorescence *in situ* Hybridization

For immuno-FISH, cells were fixed in 4% PFA, then permeabilized in 0.5% Triton X-100, blocked with 4% BSA in phosphate buffered saline with Tween 20 (PBST), and incubated with primary antibodies at 37°C overnight and secondary antibodies for 45 min. After washing in PBST, cells were again fixed with 2% formaldehyde for 10 min to fix antibodies prior to denaturing FISH steps. Cells were pre-equilibrated in 2 × SSC and treated with RNase (100 μg/ml) at 37°C for 1 h. After washing in 2 × SSC, cells were dehydrated with a 70, 90, and 100% ethanol series. Slides were then submerged in pre-heated (80°C) 70% formamide/2 × SSC (pH 7.5) for 20 min, followed by another ethanol dehydration series. Slides were air dried and hybridized to genomic BAC/fosmid probes labeled with digoxigenin (Table 2). Hybridizations were incubated at 37°C overnight in a humid chamber and then washed in 2 × SSC at 45°C, followed by 0.1 × SSC at 60°C. Slides were then pre-equilibrated in 4 × SSC and 0.1% Tween-20 and blocked with BSA before incubating with fluorochrome-conjugated digoxigenin antibodies (Jackson ImmunoResearch Laboratories). DNA was visualized with 4,6-diamidino-2 phenylindole, dihydrochloride (DAPI), and coverslips were mounted in Fluoromount-G (EM Sciences).

**TABLE 2:** List of FISH probes and their mm9 genome build coordinates.

Gene	Probe name	Chr	Start	End
*Fmo3*	WI1-2752G17	chr1	164883441	164928036
*Irs1*	WI1-0392I10	chr1	82257681	82301126
*Irs1*	WI1-1588J17	chr1	82297669	82336033
*Vldlr*	WI1-0872C17	chr19	27304574	27344776
*Msmo1*	RP24-171D10	chr8	67115518	67275946

For FISH on liver sections, paraffin-embedded sections of mouse livers were cut from tissue blocks at 6 μm thickness and mounted on slides. Sections on slides were heated at 60°C for 20 min and washed four times in xylene each for 10 min to remove the paraffin wax, followed by rehydration through an ethanol series. Samples were then microwaved for 20 min in 0.1 M citrate buffer (pH 6.0), cooled, then rinsed, and stored in dH_2_O prior to dehydration through ethanol series as above. Slides were then submerged in pre-heated (80°C) 70% formamide/2 × SSC (pH 7.5) for 20 min, followed by another ethanol dehydration series. Slides were air dried and hybridized to digoxigenin-labeled genomic fosmid clones and processed further as described above.

### Microscopy and Analysis of Gene Position

To quantify the 3D position of FISH-labeled genomic loci within the nucleus, cell images were first taken as z-stacks on a widefield microscope (Nikon TE-2000 microscope equipped with a 1.45 NA 100 × objective, Sedat quad filter set, PIFOC Z-axis focus drive from Physik Instruments, and CoolSnapHQ High Speed Monochrome CCD camera from Photometrics, all run by MetaMorph image acquisition software) with a 0.2 μm spacing in z. Images of Alexa 488-labeled loci and DAPI-labeled nuclei were deconvolved using AutoQuant X (Media Cybernetics, United Kingdom).

The z stacks containing the alleles in focus were manually thresholded, and the distance of the locus to the nearest point on the nuclear periphery labeled with antibodies to Lamin A/C (rabbit polyclonal 3262) (Schirmer et al., 2001) was scored using ImageJ. Only nuclei with three or more labeled alleles were used for analysis in order to be able to account for allelic exclusion type of phenotypes, if any. Statistics were performed using Mann–Whitney test (2 groups) or Kruskal–Wallis ANOVA (> 2 groups) for non-parametric data. Data are presented as scatters overlaid with the median and interquartile range and taken as statistically significant at *p* < 0.05.

## Results

### DamID on Freshly Isolated Primary Mouse Hepatocytes

**FIGURE 1:**
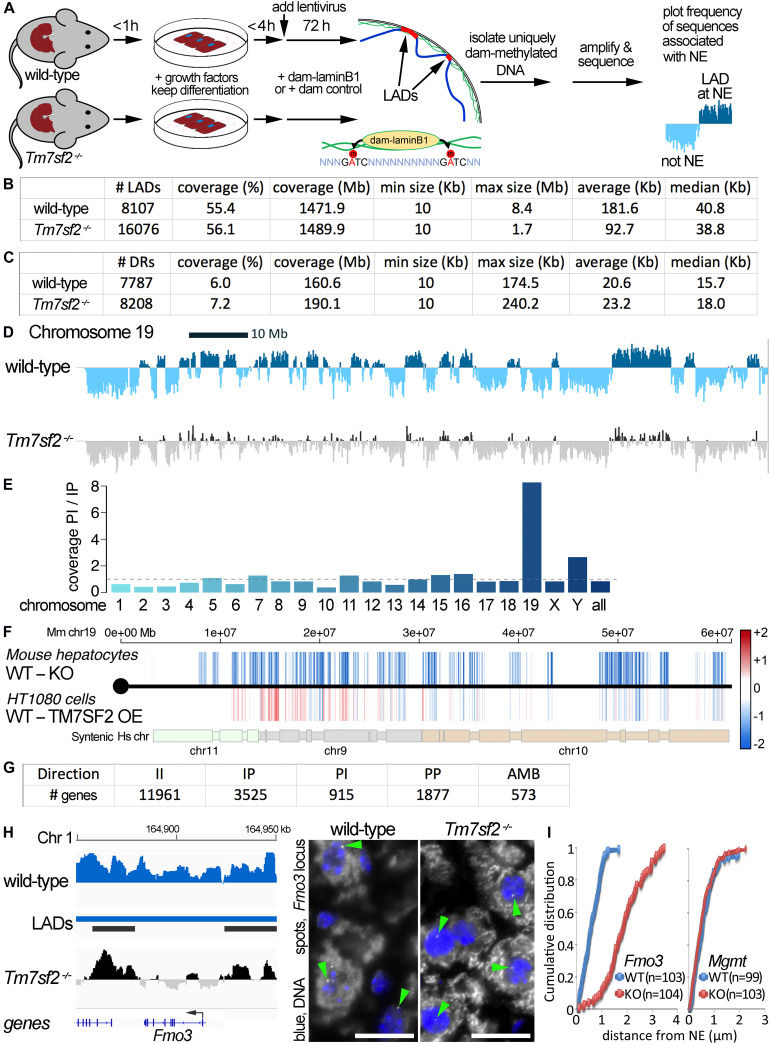
DamID mapping reveals genes dependent on Tm7sf2^–/–^ for nuclear position. **(A)** DamID approach. Livers were recovered from WT and *Tm7sf2*-KO mice and primary hepatocytes were isolated within 1 h with hormones and growth factors to maintain the differentiation state. After 4 h, cells had recovered sufficiently, achieving normal hepatocyte morphology, to be infected with lentiviruses for expression of DamID constructs—either Dam-LaminB1 fusion or a dam unfused control. Over the next 72 h, the Dam-LaminB1 confers unique methylation to the DNA in contact with the nuclear periphery that can then be isolated, sequenced, and the frequency of association with the lamina plotted. The DNA regions labeled by Dam-LaminB1 appear above the line and are referred to as LADs. **(B)** DamID metrics. Total numbers of LADs in WT and Tm7sf2-KO cells, the % coverage of the genome in LADs, the total Mb of the genome in LADs, and the minimum, maximum, average, and median size of individual LADs. **(C)** List of differential region (DR) and gene metrics. Metrics are given for the total number of DRs identified in WT and *Tm7sf2* KO liver cells, the percent of all genome covered, their total amount in the genome in Mb, and their minimum, maximum, average, and median size. **(D)** Mouse chromosome 19 DamID trace comparing cells from WT and *Tm7sf2*-KO livers. Signal plotted above the midline indicates association with the nuclear periphery, and signal below the line indicates internal nuclear localization. **(E)** Bar plot of the ratios of the proportion of individual chromosomes in PI versus IP regions. **(F)** Mouse (Mm) chromosome 19 map comparing DRs from the mouse *Tm7sf2* KO against syntenic DRs from a study in human HT1080 cells overexpressing (OE) human TM7SF2. PI regions are shown in blue, and IP regions in red using a color gradient based on the fold intensity of signal change from DamID maps. Synteny between Mm chromosome 19 and human chromosomes 9, 10, and 11 is indicated at the bottom. Syntenic blocks were obtained from Cinteny (https://cinteny.cchmc.org), whereas individual Mm DR coordinates were converted to human coordinates using the LiftOver tool at UCSC browser (https://genome.ucsc.edu/cgi-bin/hgLiftOver). **(G)** List of numbers of genes in DRs broken down by type of change (II, IP, PI, PP as defined in the text or AMB for ambiguous) based on mouse genes present in the Affymetrix MOE430 v2.0 microarray (hence fewer genes being represented than the total in the mouse genome). **(H)** On the left, *Fmo3* DamID trace as in **(D)**. The position of the *Fmo3* gene is shown against the DamID trace, and the start/orientation is indicated by an arrow. On the right, confirmation of DamID results by fluorescence *in situ* hybridization (FISH) on mouse liver tissue using probes for the *Fmo3* gene. In the images, the nuclear area is defined by DAPI staining in blue, and the white spots highlighted by green arrowheads within the nuclei are the *Fmo3* gene, which can be observed to be at the edge of the nucleus in the WT, but has moved away from the edge in the KO livers. Scale bar: 50 μm. **(I)** Quantification of the position of the *Fmo3* gene with respect to its distance from the NE in the FISH images obtained from mouse liver tissues. The cumulative distribution is plotted so that it can be observed that nearly all cells within WT liver have *Fmo3* within 1 μm from the NE, whereas in the KO, nearly every cell has it > 1 μm from the NE. As a negative control, the position of the *Mgmt* gene was similarly quantified in liver sections from the same mice because this gene did not reposition according to the DamID data.

DamID was used to globally identify genome regions at the nuclear periphery in livers of *WT* and *Tm7sf2^–/–^* mice (Figure 1A). DamID fuses a bacterial dam methylase to LaminB1 so that all DNA in contact with the NE becomes uniquely labeled and can then be isolated and sequenced. The genome regions at the NE are thus referred to as LADs. Due to ineffective viral penetrance into intact liver tissues in living animals, lentiviral vectors expressing Dam methylase constructs were transduced onto freshly isolated hepatocytes as previously described, obtaining regularly 30–40% transduction efficiencies (Gatticchi et al., 2019). To maintain genome organization as close to the intact tissue as possible before transduction, the time taken from anesthesia to cell plating was kept between 1 and 1.5 h, and lentiviral transduction occurred after only 2–4 h of culture (Nguyen et al., 2002; Selden et al., 2007). By this time, hepatocytes visibly recover from the stress of isolation, having a good refractive index and round-shaped cytoplasm, clear and distinct nuclei (mostly binucleated), and the cuboidal morphology observed in liver tissue organization (Gatticchi et al., 2019). As isolated hepatocytes are prone to lose their defined differentiation state progressively over time in culture when using normal media, the culture medium was optimized to maintain liver-specific functions by removing serum and supplementing with insulin, dexamethasone, and EGF, all of which help in slowing the de-differentiation process (Michalopoulos et al., 2003; Vinken et al., 2006; Godoy et al., 2010; Magner et al., 2013). Importantly, the characteristic liver morphology is fully maintained until the time of harvesting the dam-methylated DNA (Gatticchi et al., 2019). EGF also inhibits apoptosis in the liver cells (Ove et al., 1989; Musallam et al., 2001; Bowen et al., 2014; Vinken et al., 2014), thus avoiding apoptotic DNA fragments competing in the adaptor ligation and PCR amplification steps that enrich for the dam-methylated sequences prior to sequencing. After overnight transduction, fresh media were replaced, and cells were cultured for an additional 48 h to allow the dam fusions to methylate DNA. Thus, cells were no more than 60 h from isolation by the time that genomic DNA was recovered. The genomic DNA from four mice was pooled for the main experiment, resulting in four animals tested in each experimental condition. DNA was prepared for DamID-Seq as previously described (Robson and Schirmer, 2016; Gatticchi et al., 2019).

The enriched dam-methylated DNA was sequenced at a depth of 1.9 × (63.2 M usable reads) and 1.1 × (33.8 M usable reads) genome coverage, respectively, for WT and *Tm7sf2^–/–^* mice (all sequence reads deposited at GEO repository, ID GSE151044). LAD coordinates were then determined from DamID profiles (representing the ratio of Dam-LaminB1 to Dam alone signal) using the circular binary segmentation (CBS) algorithm found in the BioConductor/R package DNAcopy (Seshan and Olshen, 2016). This identified 8,107 LADs in WT livers versus 16,076 LADs in the KO livers with respective average sizes of 182 and 93 kb that both yielded ∼55% genome coverage (Figure 1B; Supplementary Table 1). We then compared the LADs between the WT and KO data to identify DRs uniquely present at the NE of WT or KO livers (Figure 1C). For simplicity, DRs at the periphery (NE) in WT and interior in the KO are referred to as PI, and DRs that are in the interior in the WT and at the NE in the KO are referred to as IP, whereas unchanged regions are II and PP.

There was a striking effect on chromosome 19 that exhibited a marked loss of NE associations according to the DamID profiles (Figure 1D). Compared with other chromosomes, the proportion of the chromosome in PI regions exceeded that in IP regions by 8-fold (Figure 1E). As we previously performed DamID on HT1080 human fibroblast cells overexpressing Tm7sf2 (de las Heras et al., 2017) (NCBI GEO GSE87228), we decided to compare the overexpression with the KO data for chromosome 19 despite its using a different cell type and organism. The intensity difference in the DRs between the WT and KO mice was plotted according to position across mouse chromosome 19 on the top, whereas on the bottom, the intensity difference of the DRs was plotted for HT1080 WT and TM7SF2 overexpression for the syntenic human chromosome regions (Figure 1F; Supplementary Table 2). Many of the PI DRs of the mouse liver *Tm7sf2* KO increased in signal in the HT1080 cells overexpressing TM7SF2, indicating a repositioning toward the NE (IP), particularly in the first half of the chromosome. There were 15 genes identified within these regions that reposition in opposite directions in the *Tm7sf2* mouse KO compared with the TM7SF2 HT1080 overexpression. Interestingly, while most of them are not normally expressed above background levels in adult liver, none of these were differentially expressed. This suggests that one function of TM7SF2 is to repress non-liver genes. Of note, many of these genes had links to metabolic disease and diabetes (Supplementary Table 3).

A broader DR analysis over the entire genome revealed 915 PI genes and 3,525 IP genes (Figure 1G). Thus, the KO of this one NE protein had a profound effect on radial genome organization. The most highly upregulated gene in the *Tm7sf2* KO mouse (Bennati et al., 2008) was PI gene *Fmo3* (Figure 1H), so this was chosen to confirm DamID results and Tm7sf2 dependence by FISH. *Fmo3* encodes flavin-containing monooxygenase 3, an enzyme involved in xenobiotic metabolism by 1N-oxygenating primary aliphatic alkylamines. The loss of *Fmo3* NE positioning in the *Tm7sf2^–/–^* mouse liver was first directly confirmed by FISH in tissue sections cut from intact livers (Figure 1H). Quantification of the distance to the NE in 100 cells revealed a strong shift between the WT and KO livers (Figure 1I). In contrast, the *Mgmt* gene that was called as PP by the DamID data tended to be at the nuclear periphery in both WT and KO livers by the FISH analysis (Figure 1I).

### Correlations Between Positional Changes and Expression Changes Highlight Functional Pathways Important for Liver

**FIGURE 2:**
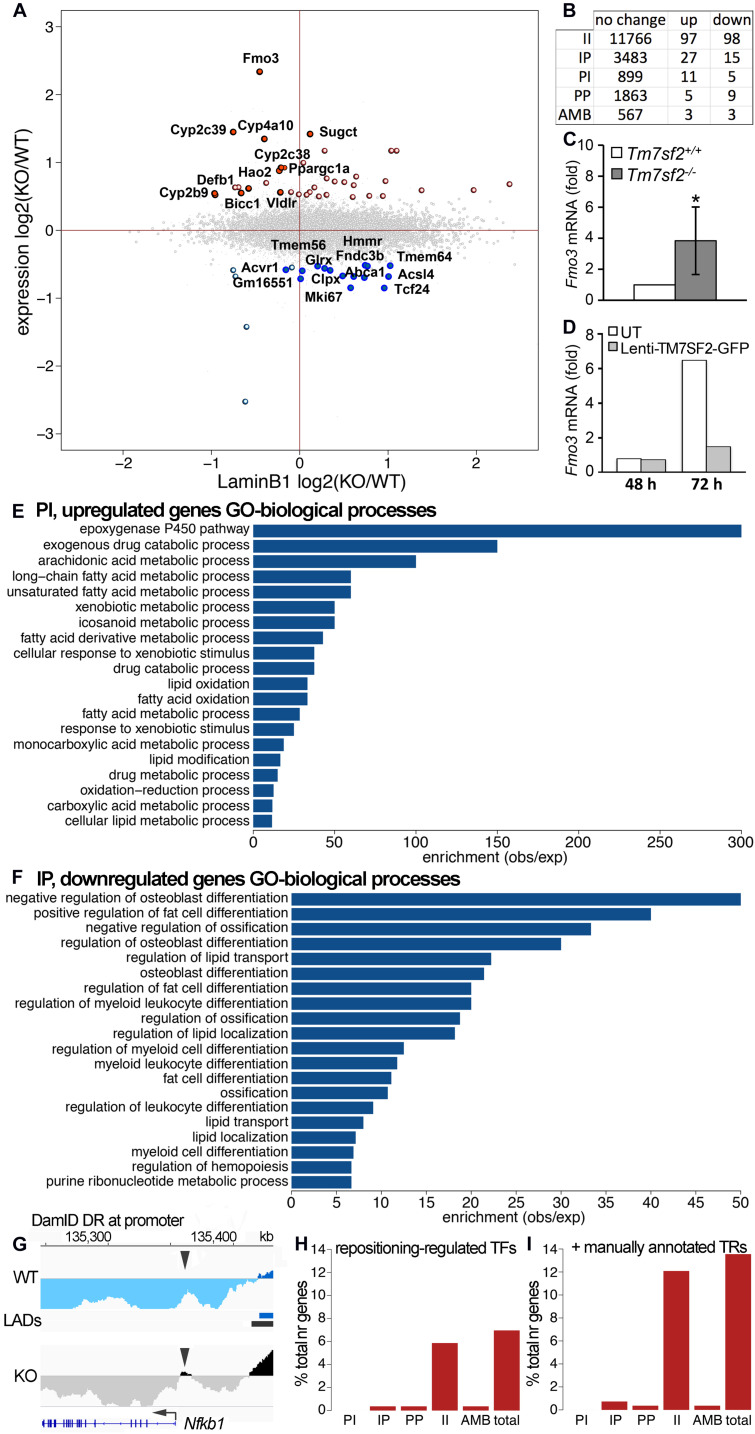
Tm7sf2-dependent nuclear positioning affects expression of some genes. **(A)** Scatterplot of expression changes against DamID signal changes averaged over a 100 kb region centered in each gene. PI genes tend to be in the left half, and IP genes in the right half. Upregulated genes are in the top half (strong red for PI, pale red for IP), and downregulated in the bottom half (strong blue for IP, pale blue for PI). The stronger colors denote that repositioning and expression changes occurred in the expected direction (i.e., NE release and upregulation and NE recruitment and downregulation), whereas the pale colors indicate the changes that occurred in the opposite direction. **(B)** Combined gene expression and positioning stats. **(C)** Confirmation of the *Fmo3* change in expression by qRT-PCR for the levels of *Fmo3* mRNA relative to *Hprt* in livers of *Tm7sf2^+/+^* (*n* = 5) and *Tm7sf2^–/–^* (*n* = 5) mice fed with a chow diet. Data are given as mean ± SD. *p < 0.05 for differences with respect to *Tm7sf2^+/+^* mice; unpaired two-tailed Student’s *t*-test. **(D)** Restoration of normal *Fmo3* regulation by rescue of Tm7sf2 expression in the KO primary isolated hepatocytes. *Tm7sf2^–/–^* primary hepatocytes were transduced for 24 h with lentiviral vector encoding for human *TM7SF2*-*GFP*, and RNA was isolated 48 and 72 h after initial transduction. *Fmo3* mRNA levels were assessed by qRT-PCR compared with the housekeeping gene *Hprt*. UT, untransduced *Tm7sf2^–/–^* cells. **(E)** topGO analysis bar chart for PI genes, released from the NE in *Tm7sf2*-KO hepatocytes. GO ID designations appearing in the graph from top to bottom are GO:0019373, GO:0042738, GO:0019369, GO:0001676, GO:0033559, GO:0006805, GO:0006690, GO:1901568, GO:0071466, GO:0042737, GO:0034440, GO:0019395, GO:0006631, GO:0009410, GO:0032787, GO:0030258, GO:0017144, GO:0055114, GO:0019752, and GO:0044255. See figure for GO term descriptions. **(F)** topGO analysis bar chart for IP genes, recruited to the NE in *Tm7sf2*-KO hepatocytes. GO ID designations appearing in the graph from top to bottom are GO:0045668, GO:0045600, GO:0030279, GO:0045667, GO:0032368, GO:0001649, GO:0045598, GO:0002761, GO:0030278, GO:1905952, GO:0045637, GO:0002573, GO:0045444, GO:0001503, GO:1902105, GO:0006869, GO:0010876, GO:0030099, GO:1903706, and GO:0009150. See figure for GO term descriptions. **(G)** DamID trace as in Figure 1 of the *Nfkb1* gene showing a small DR at only the promoter, but not in the gene body. Arrowheads point to the DR region at the promoter. **(H)** Proportion of indirectly regulated genes in each positional class, and total, i.e., those whose change in expression can be explained through differential expression of TFs, and not necessarily be a direct effect of repositioning, using mouse transcription factor and target data from the TRRUST v2.0 database. **(I)** Same as **(H)**, with additional transcriptional regulator (TR) data obtained from Charos et al. (2012) for Ppargc1a targets and Srebp1c targets from the TRRUST database.

Previous DamID studies have shown that a subset of repositioning genes change expression, and for this subset, there is a general trend for repositioning from the nuclear interior to the NE to be associated with repression and repositioning from the NE to the nuclear interior with activation (Pindyurin et al., 2007; Peric-Hupkes et al., 2010; Robson et al., 2016). Accordingly, we plotted the Dam-LaminB1 signal against previously published expression data from WT and *Tm7sf2^–/–^* mouse liver cells (Bennati et al., 2008). Upregulated genes are shown on the top half (strong red for PI, pale red for IP) and downregulated in the bottom half (strong blue for IP, pale blue for PI) (Figure 2A). The stronger colors denote that repositioning and expression changes occurred in the expected direction, whereas the pale colors indicate the changes that occurred in the opposite direction. For those repositioning genes that also exhibited a change in expression, the expected directional trends could be observed for PI genes with 69% exhibiting an increase in expression, but for IP genes, only 36% exhibited decreased expression. As expected, only a subset of repositioning genes also had changed expression (Figure 2B; Supplementary Table 1), possibly because repositioning acts in concert with transcriptional regulators present in the differentiated cells (Robson et al., 2016). Of the 915 PI genes, 11 exhibited a corresponding > 1.4-fold loss of repression associated with their NE release (Table 3). In the opposite direction, of the 3,525 IP genes, 15 had a > 1.4-fold loss in expression corresponding to their repositioning to the NE (Table 3).

**TABLE 3:** Genes repositioning and changing expression in *Tm7sf2^–/–^* liver cells.

Gene	Position	Δexp	Liver link
*Fmo3*	PI	Up	Hepatic metabolism, diabetes, insulin signaling
*Hao2*	PI	Up	Fatty acid metabolism in the liver and kidney
*Cyp4a10*	PI	Up	Fatty acid metabolism in the liver
*Ppargc1a*	PI	Up	Fatty acid metabolism in the liver; NAFLD; IRS1:IRS2 regulator
*Cyp2b9*	PI	Up	Liver expression; xenobiotic metabolism
*Defb1*	PI	Up	Prominent expression in hepatocytes; induced during cholestasis
*Bicc1*	PI	Up	Liver fibrosis
*Sugct*	PI	Up	Lipid metabolism, high liver expression
*Vldlr*	PI	Up	Cholesterol metabolism; hepatic steatosis
*Cyp2c38*	PI	Up	Fatty acid metabolism; xenobiotic metabolism
*Cyp2c39*	PI	Up	Fatty acid metabolism; xenobiotic metabolism; liver fibrosis
*Tcf24*	IP	Down	
*Apcs*	IP	Down	Liver fibrosis
*Acvr1*	IP	Down	Regulator of hepcidin production in hepatocytes
*Fndc3b*	IP	Down	Target of miR-143, associated with NAFLD
*Tmem56*	IP	Down	Predominant expression in the liver
*Tmem64*	IP	Down	Regulator of Ca^2+^ in osteoclastogenesis, low bone mineral density linked to NAFLD
*Abca1*	IP	Down	Cholesterol homeostasis; insulin signaling and lipogenesis in the liver
*Mki67*	IP	Down	
*Clpx*	IP	Down	Predominant expression in the liver
*Gm16551*	IP	Down	Regulator of Srebp1c and lipogenesis in the liver
*Acsl4*	IP	Down	Fatty acid metabolism; NAFLD
*Hmmr*	IP	Down	Liver fibrosis; hepatic wound healing and regeneration
*Glrx*	IP	Down	Liver metabolism; deficiency causes fatty liver and dyslipidemia
*Lifr*	IP	Down	Liver regeneration; fibrosis
*Mal2*	IP	Down	Lipid raft protein essential for transcytosis in liver cells

*NAFLD, nonalcoholic fatty liver disease.*

The largest expression change in the KO mouse liver (besides *Tm7sf2* itself) was in the *Fmo3* gene noted above to be important for xenobiotic metabolism. *Fmo3* is normally at the NE in the liver and loses this positioning in the *Tm7sf2^–/–^* livers (Figures 1H,I). This expression change in the KO liver was confirmed also in the primary hepatocytes by qPCR, revealing an increase in expression of greater than 3-fold (Figure 2C). The higher *Fmo3* levels seem to drop transiently upon isolation of the cells from KO liver; however, they recover by 72 h. As this was sufficient time to achieve expression from lentivirus transduction, we were able to confirm that exogenous expression of human TM7SF2 in the freshly isolated primary KO liver cells can reverse the increased expression associated with *Fmo3* release (Figure 2D).

The *Fmo3* function in xenobiotic metabolism raised the question of whether other genes released and upregulated by loss of the Tm7sf2 gene repositioning function have similar functions that require additional layers of regulation from the NE, e.g., whether its targets have shared pathways or biological functions. GO analysis for biological functions further revealed that Tm7sf2-regulated PI genes that increased in expression > 1.4-fold tended to have functions associated with the epoxygenase P450 pathway at roughly 300-fold enrichment (Figure 2E). Other highly enriched categories include exogenous drug catabolic processes, arachidonic acid metabolic processes, and long-chain or unsaturated fatty acid metabolic processes enriched, respectively, by roughly 150-, 100-, and 50-fold. This all makes sense together as several Cyp2 and Cyp4 subfamily members of the P450 family of enzymes were upregulated concomitant with the release of the genes encoding them from the NE (Table 3), and these subfamilies play roles in fatty acid metabolism, functioning on specific fatty acid substrates, such as the omega-3 eicosanoids and the omega-6 arachidonic acid. This is in contrast to the Cyp3, Cyp7, Cyp8, Cyp27, Cp39, Cyp46, and Cyp51 subfamilies that have more liver-related functions, such as in cholesterol metabolism and in bile acid synthesis (see^4^ for a table of different Cyp family member functions). In the other direction, Tm7sf2-regulated IP genes that decreased in expression > 1.4-fold tended to have GO-functional categories associated with aspects of tissue differentiation (Figure 2F). Specifically the most enriched GO-process at ∼50-fold was negative regulation of osteoblast differentiation i.e. genes that are normally active in the liver to repress the alternative osteoblast differentiation pathway that somehow get recruited to the NE and repressed in the absence of Tm7sf2 function. The next most enriched was positive regulation of fat cell differentiation, and in this case, the opposite would apply because liver differentiates fat cells in addition to hepatocytes. This is consistent with previous studies suggesting that a role of tissue-specific genome organization is to strengthen repression of alternative differentiation pathways (Robson et al., 2016; de las Heras et al., 2017). Importantly, all but two of the genes that repositioned in both directions with appropriate expression changes had liver-related functions (*Tcf24* and *Mki67*; see Table 3).

Interestingly, a previous study exogenously overexpressing Tm7sf2 in the non-liver HT1080 human fibroblast cancer cell line tended to similarly alter the position and expression of liver genes (de las Heras et al., 2017); however, the specific genes changing tended to be different from those altered in the *Tm7sf2^–/–^* livers. For example, *Fmo3* exhibited a strong expression change corresponding to its repositioning in the mouse liver (Figures 1I, 2C), whereas in the HT1080 cells, *Fmo3* gene position and expression were both unaffected. Similarly, while Cyp2c38, Cyp2c39, and Cyp4a10 were all released from the NE and activated in the *Tm7sf2^–/–^* livers, none of the cytochrome P450 Cyp2c or Cyp4a genes were repositioned or altered in expression in the HT1080 fibroblasts. Interestingly, the *Acsl4* gene was strongly affected here in liver cells, and the similarly important *Acsl5* gene was not, whereas in the HT1080 cells overexpressing TM7SF2, the *Acsl5* gene was strongly affected and not the *Acsl4* gene. This shows specificity for TM7SF2 targets, but whether the differences reflect the differences in the milieu of transcriptional regulators between fibroblast and liver cells or between human and mouse cells or something else remains unclear.

Finally, in addition to the genes mentioned above with corresponding repositioning and expression changes, 15 genes changed position with expression changes in the opposite from the expected direction, and 102 additional genes changed expression in the *Tm7sf2^–/–^* mouse livers though the genes encoding them did not reposition. Some of this might potentially be explained by regulatory elements, such as enhancers being released to act on internal genes as we previously demonstrated in lymphocyte activation (Robson et al., 2017), but this could not be investigated without also doing Hi-C here. Promoters similarly could reposition independent of the gene body and so not be called by the algorithm determining DRs as was observed for the *Nfkb1* promoter (Figure 2G). Another possibility is that some of the genes changing expression and position might encode TFs that could have secondary effects on these other genes. Because TF factors are processive, a small expression change can be amplified for its targets; therefore, instead of using the > 1.4-fold expression change used above, we selected for changes that were statistically significant with a p-value FDR < 0.05 regardless of the fold change. This increased the number of genes changing expression from 143 to 261, among which were 29 TFs according to the TRRUST database of TFs (Table 4, top section). Searching for their known targets among the 117 genes with > 1.4-fold expression changes in the *Tm7sf2^–/–^* mouse livers that either did not reposition or moved in the opposite from the expected direction for the expression change could account for only 21 of the 117 genes in this category (Figure 2H). Some well-known TRRUST database TFs, such as Jun and Ctnnb1, had many known targets listed, but 21 of the 29 TFs that changed expression have less than 15 known targets in this database, suggesting that the results in Figure 2H are underestimated. Checking the genes with altered regulation in the KO for GO terms associated with transcriptional regulation revealed additional 9 genes that could potentially account for such secondary effects (Table 4, middle section). We also manually checked the literature for Ppargc1a transcriptional targets as this PI gene had increased expression in the KO, finding a study that identified binding sites within the promoter regions of 558 genes in the human liver HepG2 cancer cell line (Charos et al., 2012). This list contained 12 targets among those changing expression in the KO by > 1.4-fold, all of which were among the group of II genes and 11 of which had not been identified in the TRRUST database analysis. Similarly, at least three other genes under Tm7sf2 positional regulation that did not appear in the TRRUST database have reported transcription regulation functions that could contribute secondary effects on the expression of non-repositioning genes altered in expression in the *Tm7sf2*-KO, but have too little known about their function to perform a useful bioinformatic analysis. One of these is transcription factor 24 (Tcf24) that was one of the two genes without a clear liver function, likely related to the fact that this TF has only two papers that mention it in lists. Similarly, Bicc1, though an RNA-binding protein, has been shown to affect gene expression by antagonizing miRNA activity (Tran et al., 2010) and was separately found in an expression profiling study to be altered in hepatic steatosis-driven fibrosis (Ramnath et al., 2018). Finally, little is known about Gm16551 function, but it has been found to be a regulator of the TF Srebp1c and lipogenesis in the liver (Yang et al., 2016). Accordingly, we searched our list of 117 genes with unaccounted for expression changes in the KO for Srebp1c targets, finding 6 targets. Thus, adding the manual analysis for just two transcriptional regulators for which more liver-specific transcription data were available nearly doubled the number of targets identified (Figure 2I).

**TABLE 4:** Transcriptional regulators.

TF gene name	Pos	Total targets	Δexp targets	Δexp non-repositioning targets
TRRUST*				
*Ahr*	IP	22	2	*Gsta1 Smoc2*
*Arntl*	II	12	1	*Dbp*
*Bcl6*	II	18	2	*Bcl6 Cdkn1a*
*Ccnd1*	II	1	0	
*Cry1*	IP	1	0	
*Ctnnb1*	II	62	1	*Tbx3*
*Dbp*	II	3	0	
*Foxa1*	II	10	0	
*Foxa3*	II	1	0	
*Foxo3*	II	9	0	
*Hhex*	II	1	0	
*Htatip2*	II	3	0	
*Jun*	II	131	3	*Pdk1 Ctse Jun*
*Klf1*	II	5	0	
*Mapk14*	II	1	0	
*Nfil3*	II	10	1	*Cyp7a1*
*Nr0b2*	II	19	4	*Nr5a2 Cyp7a1 Ppargc1a Cdkn1a*
*Nr1d2*	II	1	0	
*Nr1i2*	II	12	3	*Cd36 Ppara Cdkn1a*
*Nr5a2*	II	18	1	*Cyp7a1*
*Onecut1*	II	8	1	*Slc2a2*
*Parp1*	II	11	0	
*Ppara*	II	46	5	*Cyp7a1 Cd36 Retsat Gys2 Pxmp4*
*Ppargc1a*	PI	20	2	*Apoa4 Ppara*
*Ppargc1b*	II	3	0	
*Sfpq*	II	1	0	
*Tbx3*	II	10	2	*Onecut1 Cdkn1a*
*Tef*	II	1	0	
*Xbp1*	II	13	0	
+ GO:TR**				
*Fam129b*	II	–	–	–
*Scand1*	II	–	–	–
*Rnf6*	II	–	–	–
*Atoh8*	II	–	–	–
*Morf4l2*	II	–	–	–
*Hist1h1c*	II	–	–	–
*Ube2l3*	II	–	–	–
*H2*-*Ab1*	II	–	–	–
*Lpin2*	II	–	–	–
**Manually annotated**				
***Ppargc1a	PI	558	12	*Slc20a1 Dedd2 Lpin1 Pank1 Pik3ap1 Pxmp4 Nr0b2 Hsph1 Morf4l2 Hsp90aa1 Dusp1 Syvn*1
****Gm16551	IP	36	6	*Acss2 Nr0b2 Gck Cdkn1a Scd1 Elovl3*
*Tcf24*	IP	–	–	–

*First set limited to information available at TRRUST database of transcription factors.**Additional transcriptional regulators based on Gene Ontology (GO) search.***Data from Charos et al. (2012) Genome Res on Ppargc1a targets in liver HepG2 cells.****Targets shown are targets of Srebf1 (TRRUST database), which is known to be regulated by Gm16551 and contributing to adipogenesis in the liver, though the specific transcriptional targets of Gm16551 are unknown.

### Cholesterol Homeostasis Is Indirectly Affected by the Loss of *Tm7sf2*

**FIGURE 3:**
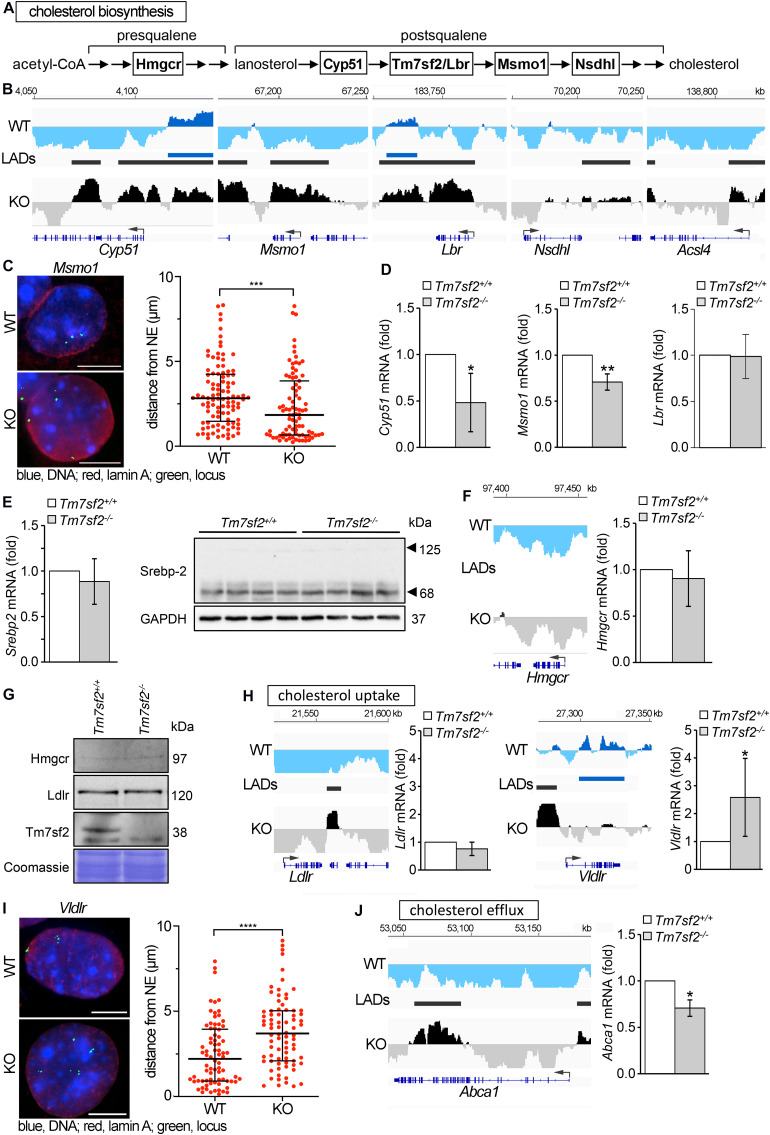
Genes affecting cholesterol homeostasis are altered by Tm7sf2 function. **(A)** Sequential steps in the cholesterol biosynthesis pathway with genes investigated here highlighted. **(B)** DamID traces of *Cyp51*, *Msmo1*, *Lbr*, *Nsdhl*, and *Acsl4* comparing cells from WT and *Tm7sf2*-KO livers. Signal plotted above the line indicates association with the nuclear periphery, and signal below the line indicates internal nuclear localization. The position of the genes is shown under the DamID trace. **(C)** FISH confirmation of *Msmo1* locus repositioning on the left and quantification of distance from the NE on the right in *Tm7sf2^+/+^* and *Tm7sf2^–/–^* primary hepatocytes. ***p < 0.005 using a Mann–Whitney test. Scale bar: 10 μm. **(D)** qRT-PCR analysis for the expression levels of *Cyp51*, *Msmo1*, and *Lbr* mRNA relative to *Hprt* in livers of *Tm7sf2^+/+^* (n = 6) and *Tm7sf2^–/–^* (n = 6) mice. Data are given as mean ± SD. *p < 0.05, **p < 0.01 with respect to *Tm7sf2^+/+^* mice; unpaired two-tailed Student’s *t*-test. **(E)** On the left, qRT-PCR analysis for the expression levels of *Srebp-2* mRNA relative to *Hprt* control of Srebp-2 livers of *Tm7sf2^+/+^* (*n* = 6) and *Tm7sf2^–/–^* (*n* = 6) mice and immunoblot analysis of Srebp-2 precursor (120 kDa) and mature (68 kDa) forms in total liver homogenates of *Tm7sf2^+/+^* (*n* = 4) and *Tm7sf2^–/–^* (*n* = 4) mice. Data are given as mean ± SD with no significant differences detected. On the right, Western blot for Srebp-2 protein levels using GAPDH as a loading control. A blot with representative samples is shown. **(F)** *Hmgcr* DamID trace as above and qRT-PCR analysis for expression levels as above with no significant differences detected. **(G)** The absence of Tm7sf2 and lack of a change for Srebp-2 target gene products Hmgcr and Ldlr were also confirmed at the protein level by immunoblot analysis on total membranes isolated from liver tissue of *Tm7sf2^+/+^* (*n* = 4) and *Tm7sf2^–/–^* (*n* = 4) mice. **(H)** DamID traces of cholesterol uptake genes *Ldlr* and *Vldlr* and qRT-PCR analysis of *Ldlr* and *Vldlr* and statistics as above. Expression levels are relative to *Hprt* in livers of *Tm7sf2^+/+^* (*n* = 6) and *Tm7sf2^–/–^* (*n* = 6) mice. **(I)** FISH confirmation and quantification of *Vldlr* in primary hepatocytes as in **(C)**, ****p < 0.001. Scale bar: 10 μm. **(J)** DamID trace of cholesterol efflux gene *Abca1* and qRT-PCR analysis of *Abca1.* Expression levels are relative to *Hprt* in livers of *Tm7sf2^+/+^* (*n* = 6) and *Tm7sf2^–/–^* (*n* = 6) mice and statistics as above.

In addition to its genome organization role, Tm7sf2 has C14 sterol reductase (C14SR) enzymatic activity that catalyzes one of the reactions of the postsqualene segment in the cholesterol biosynthesis pathway, involved in the conversion of lanosterol into cholesterol (Figure 3A). Therefore, we considered the possibility that it might additionally contribute to regulation of cholesterol biosynthesis pathways through a feedback loop by regulating other genes in the pathway. Searching the DamID data for repositioning of genes involved in cholesterol biosynthesis in the *Tm7sf2^–/–^* mice revealed several IP genes both upstream and downstream of Tm7sf2 function in the cholesterol biosynthesis pathway, including *Cyp51*, *Msmo1*, *Lbr*, and *Nsdhl* (Figure 3B), respectively, encoding lanosterol 14α-demethylase, methylsterol monooxygenase 1, LBR (a functional homolog of Tm7sf2), and NAD(P)-dependent steroid dehydrogenase-like. We confirmed *Msmo1* repositioning by FISH (Figure 3C) and tested *Cyp51*, *Msmo1*, and *Lbr* transcript levels to check if the peripheral localization was associated with their repression (Figure 3D). Consistent with their presence at the NE in the KO and internal localization in the WT, expression levels were reduced for *Cyp51* and *Msmo1*; however, LBR that also repositioned according to the DamID data was not altered in expression. There were also examples of functionally relevant genes being only partly recruited to the NE. Roughly half of the *Acsl4* locus, encoding for acyl-CoA synthetase long-chain family member 4, was recruited to the nuclear periphery (IP) in *Tm7sf2*-KO cells (Figure 3B). *Acsl4* is required for cholesteryl ester synthesis from long-chain polyunsaturated fatty acids to provide substrates for steroidogenesis (Wang et al., 2019), and attenuated *Acsl4* expression in the liver results in insulin resistance due to altered very low-density lipoprotein (VLDL) metabolism (Singh et al., 2019).

*Tm7sf2*, along with several other genes involved in cholesterol biosynthesis and metabolism, is regulated by the Srebp-2 TF. Thus, we considered the possibility that expression changes might reflect loss of Srebp-2 rather than an effect of nuclear positioning. Testing both Srebp-2 gene expression and protein levels of the 68 kDa activated form and precursor (125 kDa) revealed that neither was changed in the *Tm7sf2-*KO (Figure 3E). Furthermore, Srebp-2 targets include not only *Ldlr* and *Msmo1* but also *Hmgcr* that is not repositioned (II) and does not change expression at either the mRNA or protein levels (Figures 3F,G). The absence of Tm7sf2 and lack of a change for the Srebp-2 target Ldlr were also evaluated at the protein and mRNA levels (Figures 3G,H), despite the formation of a LAD inside *Ldlr* in *Tm7sf2*-KO cells.

Overall levels of cholesterol were not altered in *Tm7sf2-*KO mice (Bennati et al., 2008). This was thought to be due to the Tm7sf2 paralog Lbr being sufficient for this function. However, there was a failure to increase cholesterol in response to ER stress and to inflammatory stimuli in the KO mice (Bellezza et al., 2013, 2015). This could be accounted for at least in part by the reduced expression in the *Tm7sf2-*KO of the cholesterol biosynthesis genes noted above that occur late in the synthesis pathway. At the same time, we wondered if other genes involved in cholesterol uptake and efflux could be impacted. In addition to *Ldlr*, the *Vldlr* gene encoding the VLDL receptor is also involved in cholesterol uptake and, though a LAD was formed upstream, was predominantly PI in the gene body and accordingly increased expression in the KO (Figure 3H). The repositioning of *Vldlr* was additionally confirmed by FISH (Figure 3I). For cholesterol efflux, the gene encoding the ATP-binding cassette subfamily A member 1 (Abca1) was partly IP with LADs forming both upstream and in the 3′ end of the gene and a corresponding slight decrease in expression (Figure 3J). Whereas *Vldlr* moved away from the NE, *Abca1* moved to the NE, and accordingly, *Abca1* was downregulated, and *Vldlr* was upregulated (Figures 3H,J).

### *Irs1* and *Irs2* Genes Are Mispositioned With Corresponding Defects in Insulin Pathways and a Mild Diabetic Phenotype in Mice

**FIGURE 4:**
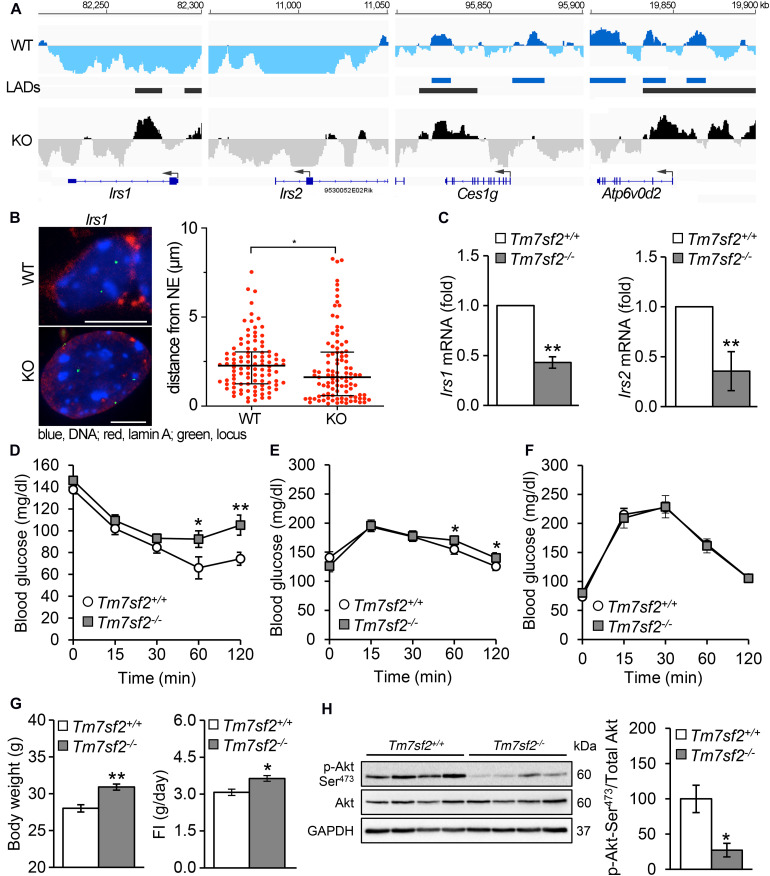
Insulin signaling pathways are defective in *Tm7sf2^–/–^* mice. **(A)** DamID traces of genes *Irs1*, *Irs2*, *Ces1g*, and *Atp6v0d2* as in Figure 3. **(B)** FISH confirmation of locus mispositioning for *Irs1* in *Tm7sf2^+/+^* and *Tm7sf2^–/–^* primary hepatocytes; quantification and statistics as in Figure 3C, *p < 0.05. Scale bar: 10 μm. **(C)** *Irs1/2* genes downregulated. qRT-PCR analysis for the expression levels of *Irs1* and *Irs2* mRNA relative to *Hprt* in livers of *Tm7sf2^+/+^* (*n* = 5) and *Tm7sf2^–/–^* (*n* = 5) mice fed with a chow diet. Data are given as mean ± SD. **p < 0.01 with respect to *Tm7sf2^+/+^* mice; unpaired two-tailed Student’s *t*-test. **(D)** Insulin tolerance tests, resting. Plasma glucose concentration during intraperitoneal insulin tolerance test (ITT) of *Tm7sf2^+/+^* (*n* = 9) and *Tm7sf2^–/–^* (*n* = 11) mice. Data are given as mean ± SEM. *p < 0.05, **p < 0.01 with respect to *Tm7sf2^+/+^* mice; unpaired two-tailed Student’s *t*-test. **(E)** Glucose tolerance tests, resting. Plasma glucose concentration during intraperitoneal glucose tolerance test (GTT) in fed condition of *Tm7sf2^+/+^* (*n* = 10) and *Tm7sf2^–/–^* (*n* = 12) mice. Statistics as in **(D)**. **(F)** Glucose tolerance test after fasting. Plasma glucose concentration during intraperitoneal glucose tolerance test (GTT) in fasted (O/N) condition of *Tm7sf2^+/+^* (*n* = 14) and *Tm7sf2^–/–^* (*n* = 9) mice. Statistics applied as in **(D)** yielded no significant differences. **(G)** Body weight of 1-year-old WT (*n* = 8) and *Tm7sf2^–/–^* (*n* = 8) mice (left) and daily food intake of WT (*n* = 19) and *Tm7sf2^–/–^* (*n* = 13) mice (right). Data are given as mean ± SEM. *p < 0.05, **p < 0.01 with respect to *Tm7sf2^+/+^* mice; unpaired two-tailed Student’s *t*-test. **(H)** Akt phosphorylation. Immunoblot analysis for phosphorylated form of Akt (Ser473) in liver lysates obtained from *Tm7sf2^+/+^* (*n* = 6) and *Tm7sf2^–/–^* (*n* = 6) mice fed on a chow diet. GAPDH was used as loading control. A blot with representative samples is shown. Graph on the right represents the mean ratio (%) ± SD of Akt Ser473 phosphorylation over total Akt levels. *p < 0.05, unpaired two-tailed Student’s *t*-test.

Several genes associated with insulin function or glucose metabolism and/or linked to diabetes were identified as repositioning concomitantly with a change in expression (Figure 4A). These include *Atp6v0d2* encoding ATPase H+ transporting lysosomal V0 subunit D2 that is involved in insulin receptor recycling, *Ces1g* encoding carboxylesterase 1G that is involved in insulin resistance, and *Irs1* and *Irs2* encoding the insulin receptor substrate 1 and insulin receptor substrate 2 proteins that regulate insulin signaling. Both *Irs1* and *Irs2* have new LADs forming proximal to their first exons in the KO (IP), and this was confirmed for *Irs1* by FISH (Figure 4B). *Ces1g* has a LAD that overlaps with the C-terminus in both the WT and KO, but in the KO it loses a LAD overlapping with the start of the gene (PI), whereas *Atp6v0d2* has a mixed pattern that is mostly PI with exons 2, 3, 5, 7, and 8 in LADs in WT liver cells that are lost in the KO though the start of the gene seems to be gaining a LAD. Despite the fact that some LADs remained on both *Ces1g* and *Atp6v0d2*, the expression data from the KO livers (Bennati et al., 2008) reveal both being moderately upregulated, consistent with their being predominantly PI. All *Atp6v0d2* probes in the microarray were in the PI 3′ region of the gene so it was not possible to distinguish from this data whether exon 1 that entered a LAD was absent; however, there is a predicted splice variant and internal promoter (XM_017320194) at the 3′ PI regions.

We decided to focus on the two Irs genes because mutations in *Irs1* are linked to type II diabetes and susceptibility to insulin resistance (Laakso et al., 1994). Irs1 is a signaling adaptor that is phosphorylated by insulin receptor kinase. Phosphorylation of Irs1 prevents its association with the insulin receptor and thus can inhibit insulin action and result in insulin resistance. *Irs2* is a paralog of *Irs1* encoding insulin receptor substrate-2, which is similarly phosphorylated by insulin receptor kinase and linked to diabetes (Almind et al., 1999; Mammarella et al., 2000), but additionally has haplotypes associated with severe obesity (Lautier et al., 2003). The drop in expression for both *Irs1* and *Irs2* was confirmed in the KO livers (Figure 4C). As might be expected from their function in insulin pathways, the *Tm7sf2^–/–^* animals exhibited a mild insulin resistance phenotype. WT animals cleared blood glucose more efficiently than the KOs, particularly at later timepoints, for both insulin tolerance tests (Figure 4D) and glucose tolerance tests (Figure 4E) under normal conditions. However, interestingly, in fasted animals for glucose tolerance tests, a difference was no longer detectable (Figure 4F). Consistent also with the obesity linkage to *Irs2* mutations (Lautier et al., 2003), the mice exhibited some weight gain (Figure 4G).

Akt is a kinase that plays a central role in regulating insulin receptor signaling downstream of *Irs1* and *Irs2*, as well as many other components of insulin pathways, and it is phosphorylated in its activated state (Alessi et al., 1996, 1997; Yecies et al., 2011). Therefore, we considered the possibility that it might work together with the repositioning changes in *Irs1* and *Irs2*, and in supporting the expression changes. Akt levels themselves were not appreciably altered in the KO, but Akt phosphorylation was strongly inhibited in *Tm7sf2*-KO liver, indicating a possible defective insulin signaling that could lead to obesity and diabetes (Figure 4H).

### Tm7sf2 Sterol Reductase Function May Contribute to Its Gene Repositioning Function

It is not known whether Tm7sf2 can directly bind chromatin/DNA as has been demonstrated to be the dominant function for its homolog LBR (Ye and Worman, 1996; Makatsori et al., 2004; Solovei et al., 2013), especially as the mapped LBR HP1-binding site is not shared by Tm7sf2. However, both LBR and Tm7sf2 have 3beta-hydroxysterol Delta(14)-reductase activity (Silve et al., 1998; Bennati et al., 2006), and this activity was suggested to be important for the pathophysiology of Greenberg skeletal dysplasia caused by LBR mutations (Waterham et al., 2003). Moreover, since the protein is in both the NE and ER (Roberti et al., 2002; Schirmer et al., 2003; Malik et al., 2010; Korfali et al., 2012; Capell-Hattam et al., 2020), it is hard to determine if its effects on genome organization are direct from the NE or indirect from the ER. Therefore, to test if the sterol reductase function is important for the gene repositioning activity, we generated point mutations in human *TM7SF2* and expressed these from lentiviral vectors in acutely isolated *Tm7sf2^–/–^* hepatocytes from the KO animals.

**FIGURE 5:**
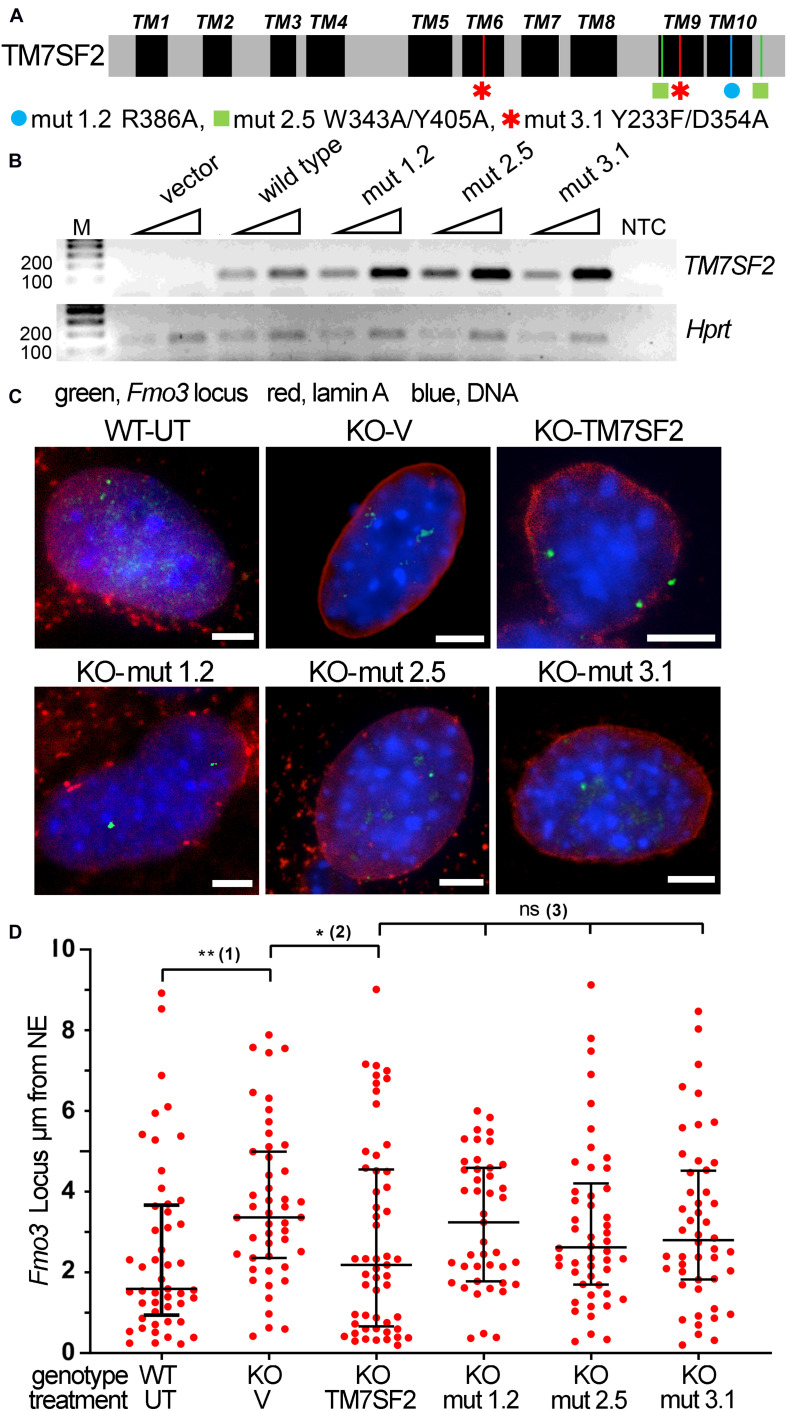
Tm7sf2 gene repositioning function requires enzymatic activity intact. **(A)** Schematic illustration of TM7SF2 linear protein sequence with transmembrane domains (TMs) highlighted in black and the position of mutations shown with a different color for each mutant. Note that although TM7SF2 was historically thought to have only 8–9 TMs (Silve et al., 1998; Roberti et al., 2002), a recent structural study (Li et al., 2015) indicated 10. **(B)** Mutant expression level. PCR analysis of WT human *TM7SF2* and its mutants in *Tm7sf2^–/–^* primary hepatocytes after 72 h of transduction. Two different amounts of starting template were used (25 and 50 ng of cDNA, triangles). Human and mouse primers were used for *TM7SF2* and *Hprt* amplification, respectively. M, DNA ladder markers. NTC, no template control. **(C)** FISH images of *Fmo3* locus from WT untransduced (WT-UT) primary mouse liver cells and rescue experiment expressing the empty vector (V) or human WT TM7SF2 and mutants in the mouse *Tm7sf2^–/–^* cells (KO). Scale bar: 5 μm. **(D)** Quantification of panel **(C)** FISH data distance from the NE. *p < 0.05, **p < 0.01, ns = non-significant, using a Kruskal–Wallis test, followed by *post hoc* Dunn correction. Statistical tests for analyzing the differential position of the Fmo3 locus were carried out against relevant controls. Compared with the WT-UT cells, the *Fmo3* locus is significantly shifted to the nuclear interior in KO-V cells that express an empty vector (1). Transduction of the human TM7SF2 vector tethers the *Fmo3* locus to the periphery compared with transduction with an empty vector (2). In contrast, the TM7SF2 mutants fail to reposition the locus to the nuclear periphery (3).

Mutants were chosen to block different aspects of the sterol reductase activity (Figure 5A), based on comparative structural studies between human TM7SF2 and its homologous MaSR1 (Li et al., 2015). Mutation 1.2, R386A, was not able to functionally complement the defective *erg-3* homolog in *Neurospora crassa* or the *erg-24* homolog in *Saccharomyces cerevisiae* (Prakash and Kasbekar, 2002). Mutation 2.5, W343A/Y405A, disrupts the NADPH binding site, whereas mutation 3.1, Y233F/D354A, alters the putative cholesterol 3-OH binding site, and both are catalytically inactive (Li et al., 2015). Note that all mutations occur in conserved residues within absolutely conserved regions between mouse and human (Supplementary Figure 1), and several of the mutations occur in the last two transmembrane domains that were not originally predicted but indicated from a recent structural study (Li et al., 2015). All transduced mutants in the KO background expressed to similar levels and even higher than the WT rescue (Figure 5B). This lends greater credibility to any rescue defects of the mutants as it was previously shown that Tm7sf2-directed chromosome repositioning increases with its expression (Zuleger et al., 2013).

FISH for the *Fmo3* gene was performed on the primary cultured liver cells at 48 h of Tm7sf2 expression (Figure 5C). Expression of the WT Tm7sf2 rescued the NE positioning, whereas this was less so for all three point mutations. Quantification of locus positioning not only confirmed the restoration of NE proximity in KO cells expressing WT Tm7sf2 compared with those transduced with an empty vector (KO-V) but also further revealed a distribution with more loci in direct proximity to the NE (Figure 5D). The distributions of all three mutants were focused between the distributions for the KO with the vector rescue and the distributions for the WT rescue; however, none reached statistical significance compared with the empty vector control (KO-V). Thus, the sterol function may indirectly contribute to the gene repositioning function, but it is not essential.

## Discussion

Nearly all of the set of 26 genes that changed both position and expression upon *Tm7sf2-*KO have clear liver functions, many of which impact on important metabolic pathways that can explain animal pathology, particularly regarding cholesterol biosynthesis and insulin regulation as demonstrated here. Why this particular set of genes is under Tm7sf2 position–expression regulation remains unclear as does the mechanism, largely because of our inability to distinguish whether additional genes with expression changes that did not reposition were related to Tm7sf2 enzymatic function or secondary and tertiary transcriptional cascades. However, the focused gene set and important effects on animal physiology from knocking down this highly liver-expressed NET are consistent with our earlier studies (Zuleger et al., 2013; Robson et al., 2016, 2017; de las Heras et al., 2017), arguing for the importance of studying genome organization in actual tissues. Moreover, the insulin and cholesterol pathways impacted by the loss of this NE protein require highly complex regulation, and accordingly, it should not be surprising that genome organization also contributes. Insulin signaling, which acts through the Akt pathway, could be influenced by alterations in the levels of total cellular cholesterol or in the composition of cholesterol-enriched membrane microdomains (Sanchez-Wandelmer et al., 2009; de Castro Fonseca et al., 2018).

However, the lower levels of phosphorylated Akt in the livers of *Tm7sf2^–/–^* mice could be explained by additional mechanisms (e.g., control in *Irs1* and *Irs2* expression), alternatively to the cholesterol biosynthesis defect. In fact, *Tm7sf2*^–/^*^–^* mice display no major differences in total or esterified cholesterol levels, with respect to WT mice (unpublished data), thanks to the presence of the vicarious C14SR activity exhibited by Lbr.

Although a different study is required to test it, we posit that the effect of Tm7sf2 loss on expression of several genes in the cholesterol uptake, biosynthesis, and efflux pathways may indicate a novel feedback loop.

Even though the regulation of Tm7sf2 subcellular distribution and its related functions remain largely unexplored, the function of Tm7sf2 in gene repositioning can potentially explain some other data previously reported for this protein. Apart from its clear enzymatic role in cholesterol biosynthesis (Bennati et al., 2008), Tm7sf2 has been shown to be important for liver regeneration (Bartoli et al., 2016), in skin papilloma formation (Bellezza et al., 2015), and affecting nuclear factor kappa B (NF-κB) activity and *TNF*α expression (Bellezza et al., 2013; Gatticchi et al., 2015). While these could follow from indirect effects on signaling pathways, from a defect in cholesterol biosynthesis, or another sterol synthesis pathway involving Tm7sf2, its gene repositioning activity could easily affect expression of genes involved in these other functions. For example, one function of gene repositioning NETs in muscle differentiation is to repress cell cycle genes by recruiting them to the NE since myotubes are non-proliferative (Robson et al., 2016), and indeed, some cell cycle genes were altered by TM7SF2 in the human HT1080 cell line (de las Heras et al., 2017), and here, one of the two genes with less clear liver-specific functions, *Mki67*, has a general function associated with cell proliferation (Izumi et al., 2018). Similarly, the reported changes in NF-κB activity and *TNF*α expression in the *Tm7sf2*-KO mice (Bellezza et al., 2013; Gatticchi et al., 2015) might be influenced by the promoter of the *Nfkb1* gene that encodes NF-κB, being under Tm7sf2 positional control, as well as two TFs (Parp1 and Jun) that act on NF-κB, and being altered in the KO. Moreover, the Akt kinase that we reported was misregulated can act on post-transcriptional regulation of NF-κB (Malanga et al., 2015).

In addition to the cholesterol biosynthesis and insulin signaling pathways that were particularly affected here, there is some indication of a function for Tm7sf2 in ER stress response pathways. We predict that the large increase in *Fmo3* gene function could potentially overwhelm the liver with production of metabolites that cannot be obtained from diet, potentially resulting in ER stress. Both *Fmo3* and its paralog *Fmo2* are under similar regulation. Several other genes that could potentially affect ER stress are also under this positional regulation, being increased in expression in the *Tm7sf2^–/–^* mice. These also include *Hao2* encoding hydroxyacid oxidase 2 and several cytochrome P450 family members. Cyp2a4, Cyp2b9, Cyp2c39, Cyp2c54, Cyp4a10, and Cyp4a14 all have multiple exons in LADs in the WT liver cells that are not in LADs in the KO cells. This generally correlates with de-repression/increased expression from 2- to 4-fold over WT.

The small subset of repositioning genes also altered in expression together with their liver specificity would seem to indicate that the repositioning is only able to influence expression when a particular milieu of transcriptional regulators is present. One hypothesis would be that a transcriptional regulator sitting on the gene interacts with Tm7sf2 and mediates the repositioning; however, there is no shared promoter element signature of the set of genes under this kind of regulation by Tm7sf2, with the exception that several are known substrates of Srebp-2. This observation, however, provides one of the strongest supports to date of the importance of genome position to gene regulation because the finding that TF Srebp-2 was unchanged confirms that the defect in gene expression for the genes undergoing repositioning cannot be due to a loss of the TF that drives them. The regulation of these genes in the cholesterol biosynthesis pathway has been studied for many years and is well understood (Hua et al., 1993; Schiavoni et al., 2010), so there is no doubt that the difference in expression must be due to the change in position between the nuclear interior and the NE, providing an additional layer of regulation mediated by the Tm7sf2 genomic function. It is important to note in this regard that the function of spatial genome organization appears to be to fine-tune gene regulation as opposed to being an on/off mechanism and as such is a branch of epigenetics.

There were also many genes that changed expression without changing position in the KO mouse. Many of these can be explained by cascading secondary and tertiary effects of transcriptional regulators where the starting changes are due to the gene repositioning altering expression in a small subset. While our analysis of existing TF data was only able to explain 13.5% of the genes with altered expression that did not have corresponding repositioning changes in the KO mouse liver cells, the fact that manual analysis of just two transcriptional regulators identified nearly as many of these as the bioinformatic analysis using the TRRUST database for 29, and that we found 41 transcriptional regulators to be altered as candidates for this function would suggest that indirect effects of transcriptional regulator may account for a significant proportion of the altered genes that appeared unlinked to the repositioning function. While the TRRUST database sets a high bar for directly confirmed regulation, the fact that it listed only 20 targets for Ppargc1a whereas 558 were found by Charos et al. (2012) when using the liver HepG2 cancer cell line further highlights the importance of determining transcriptional targets of each transcriptional regulator in the particular tissue being investigated. The lack of known targets both in general cell lines and in liver tissue for potential RNA targets of Bicc1, such as miRNAs and LncRNAs (Tran et al., 2010), further highlights deficiencies that need to be addressed before a proper assessment can be made of how many of the unaccounted expression changes are due to such secondary effects, but the finding of Bicc1 to be altered in hepatic steatosis-driven fibrosis (Ramnath et al., 2018) further underscores the relevance of such interactions and the importance of focusing associated investigations specifically in the liver system.

Nonetheless, with both its gene repositioning function and its enzymatic function, it is difficult to distinguish the relative contributions of each to different cells or organismal phenotypes. The intermediate effect on gene repositioning for the point mutations that block the sterol dehydrogenase function of Tm7sf2 might indicate that the function requires both Tm7sf2 being involved in a physical tether, and that a transcriptional regulator that is induced by a product of its sterol dehydrogenase function mediates its interaction with the locus. Indeed, the specific inhibition of C14SR activity in cultured cells leads to the accumulation of sterol intermediates able to activate nuclear receptors signaling (e.g., LXRα) (Gatticchi et al., 2017). However, since LBR and Tm7sf2 have the same dehydrogenase function, one might expect LBR to be able to still produce the cofactor, as well as to accomplish cholesterol biosynthesis. Nonetheless, this might also indicate that its substrate binding pocket is required for the interaction with this partner, which could explain all the above results. Unfortunately, the data still cannot unambiguously distinguish between a direct tether function and an indirect function requiring its enzymatic activity.

Regardless, the set of genes that changed both position and expression was nearly all important for liver function, indicating the importance of studying genome organization in tissues as opposed to cell culture systems. In our previous work in this area, we tested the overexpression of the muscle NET PLPP7/NET39 in a cancer fibroblast cell line where it altered genome organization, but only 20% of the affected genes were muscle-related genes (de las Heras et al., 2017). We tested it again in C2C12 cells, an immortalized tissue culture system recapitulating myogenesis, where 75% of genes altered by its knockdown were muscle-related genes (Robson et al., 2016). In that same study, the percentage of genes affected by two other muscle NETs WFS1 and Tmem38a that were muscle-related was 64 and 70%, respectively. Critically, the genes under Tm7sf2 radial genome positional regulation in primary mouse liver cells exhibited minimal overlap with genes under TM7SF2 regulation in the human HT1080 fibrosarcoma cancer cell line. While those that did overlap were clearly functionally relevant (Supplementary Table 3), closer to primary tissue is clearly better as here, using cells directly isolated from mouse livers, more than 90% of the genes affected had liver-related functions.

In conclusion, by showing that the Srebp-2 TF driving expression from several of the Tm7sf2 affected loci was unchanged, this study has been able to isolate the effects of gene position from other changes during differentiation that made conclusions difficult in other studies, while using a more tissue-based primary cell system. The changes were consistent with NET-directed changes in our previously published myogenesis study (Robson et al., 2016) where positioning does not turn genes on or off, but affects their expression generally 2–4-fold. Most importantly, the fact that Tm7sf2 could also alter the regulation of genes associated with liver functions in HT1080 cells, but the specific gene targets mostly differed from those in actual liver tissue with very little overlap emphasizes the importance of using actual tissues when studying genome organization and strongly argues against the use of cancer cell lines to study tissue-specific aspects of genome organization.

## Data Availability Statement

DamID data and a table integrating its analysis with the expression data are available on NCBI GEO with ID GSE151044. Raw microarray data and the subsequent processed data used in this study are available on NCBI GEO with ID GSE155762.

## Ethics Statement

The animal study was reviewed and approved by Animal Care and Use Committee of Perugia University and the Italian Ministry of Health.

## Author Contributions

LG performed most experiments and managed mice. JH performed DamID and all bioinformatics. AS performed FISH experiments in Figures 3–5. NZ initiated the study of NET47/Tm7sf2 and performed FISH experiments in Figure 1. RR generated the *Tm7sf2* knockout mouse and oversaw experiments at U. Perugia. ES wrote the manuscript and oversaw experiments at U. Edinburgh. All authors contributed to the article and approved the submitted version.

## Conflict of Interest

The authors declare that the research was conducted in the absence of any commercial or financial relationships that could be construed as a potential conflict of interest.

## Footnotes

1 http://biogps.org

2 http://www.grnpedia.org/trrust

3 http://www.geneontology.org

4 https://themedicalbiochemistrypage.org/cytochrome-p450-cyp-enzymes/

## Supplementary Material

Supplementary Table 1 Summary of DamID and transcriptome data (XLSX format). Raw data available on NCBI GEO with ID GSE151044 (DamID) and GSE155762 (transcriptome). Data column description is found in the file as a separate tab.

Supplementary Table 2 DamID signal (median) for PI regions in chr19, and their human syntenic counterparts in HT1080 cells overexpressing Tm7sf2.

Supplementary Table 3 Tissue expression and disease links for genes in chr19 that lose NE association in the livers of Tm7sf2 KO mice and gain DamID signal in human HT1080 cells overexpressing Tm7sf2.
