# Supplementary figures and images for: Tm7sf2 Disruption Alters Radial Gene Positioning in Mouse Liver Leading to Metabolic Defects and Diabetes Characteristics
Source: Front Cell Dev Biol. 2020 Nov 23;8:592573. doi: 10.3389/fcell.2020.592573 (PMC7719783; doi:10.3389/fcell.2020.592573)

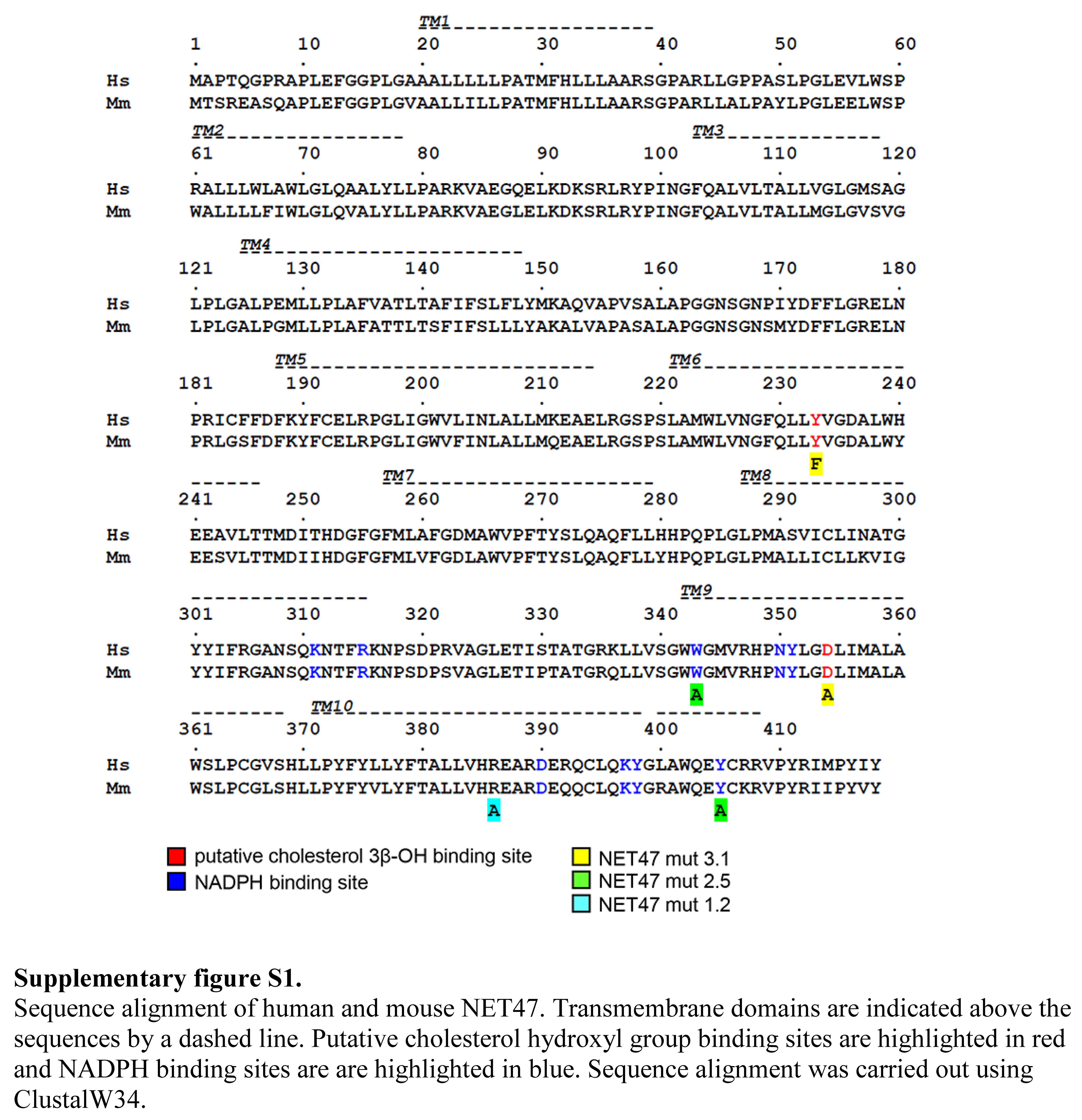

Supplement: Supplementary file 4 [file Image_1.TIF]
